# Physical and respiratory therapy in the critically ill patient with obesity: a narrative review

**DOI:** 10.3389/fmed.2024.1321692

**Published:** 2024-02-21

**Authors:** Miguel Ángel Martínez-Camacho, Robert Alexander Jones-Baro, Alberto Gómez-González, Diego Morales-Hernández, Dalia Sahian Lugo-García, Andrea Melo-Villalobos, Carlos Alberto Navarrete-Rodríguez, Josué Delgado-Camacho

**Affiliations:** ^1^Critical Care Physical Therapy Department and Post-operative Recovery and Multi-Organ Support Unit, Hospital General de México “Dr. Eduardo Liceaga,” Mexico City, Mexico; ^2^Doctorate Programme in Health Sciences, Universidad Anahuac Norte, State of Mexico, Mexico; ^3^Master’s Programme in Health Sciences, Instituto Politecnico Nacional, Mexico City, Mexico

**Keywords:** physical therapy, early mobilization, rehabilitation, critical care, obesity

## Abstract

Obesity has become increasingly prevalent in the intensive care unit, presenting a significant challenge for healthcare systems and professionals, including rehabilitation teams. Caring for critically ill patients with obesity involves addressing complex issues. Despite the well-established and safe practice of early mobilization during critical illness, in rehabilitation matters, the diverse clinical disturbances and scenarios within the obese patient population necessitate a comprehensive understanding. This includes recognizing the importance of metabolic support, both non-invasive and invasive ventilatory support, and their weaning processes as essential prerequisites. Physiotherapists, working collaboratively with a multidisciplinary team, play a crucial role in ensuring proper assessment and functional rehabilitation in the critical care setting. This review aims to provide critical insights into the key management and rehabilitation principles for obese patients in the intensive care unit.

## Introduction

Obesity is widely recognized as a global public health challenge that knows no age or gender boundaries, transcends socio-economic conditions, and silently gives rise to an epidemic that claims lives and diminishes the quality of life (QoL) for millions of individuals annually worldwide, surpassing even underweight conditions (1). The World Health Organization (WHO) defines it as “the abnormal or excessive accumulation of body fat that may have adverse health implications.” Also, it categorizes obesity based on the body mass index (BMI), which is calculated as the quotient of weight in kilograms and height in meters squared, as presented in Table 1.

**TABLE 1:** BMI according to the WHO.

Category	IMC (kg/m^2^)
Underweight	< 18.5
Normal weight	18.5–24.9
Overweight	25 a 29.9
Obesity Class I	30 a 34.9
Obesity Class II	35 a 39.9
Obesity Class III (morbid or extreme)	≥40

In 2016, an estimated total of 1.9 billion adults were afflicted with obesity, and over 650 million were overweight worldwide (1). This condition is characterized by its chronic and progressive nature, impacting the entire organism and predisposing individuals to multiple diseases across various systems. It is mediated by a state of chronic inflammation associated with an altered response of adipocytes, which disrupts the immune and metabolic state of the cell (2, 3).

Obesity’s relevance within the intensive care unit (ICU) arises from a multitude of disturbances, at various levels, which typically categorize the population as a comprehensive challenge for the staff involved in their care. Some of these are related to risk stratification for malnutrition, metabolic, respiratory and hemodynamic care, management of pharmacological interactions, transfers, hygiene, and an increased susceptibility to nosocomial infections (4, 5). The complexity and impact of obesity on clinical and therapeutic decision-making is not adequately captured by relying on a single “severity” indicator such as BMI. Similarly, anthropometric measures such as waist circumference and waist-to-hip ratio are imperfect models that maintain age, gender, and muscle mass and adipose tissue distribution biases (6, 7).

Thus, a series of methods dependent on sophisticated technology have been proposed for the quantification of total body mass components, the identification of the degree of obesity, its evaluation, the use of energy, and the study of pre-existing and/or disease-related comorbidities. Some of these methods include electrical bioimpedance, computed tomography, magnetic resonance imaging, indirect calorimetry, among others. However, these methods still lack validity and some are not widely available. International work-teams and institutions continue working and debating about which perspective adapts better through clinical relevance and impact.

**FIGURE 1:**
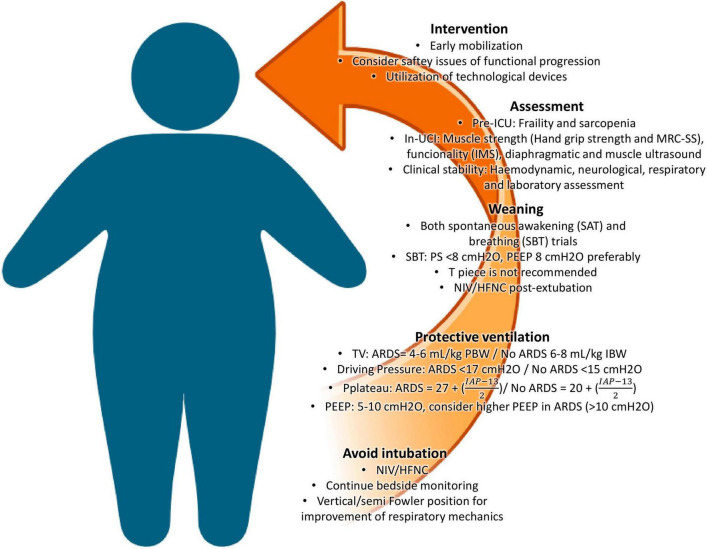
Key management and interventions for achieving better outcomes in critically ill patients with obesity. NIV: non-invasive ventilation; HFNC: high-flow nasal cannula; TV: tidal volume; PBW: predicted body weight; IBW: ideal body weight; PEEP: positive-end expiratory pressure; ARDS: acute respiratory distress syndrome; PS: pressure support; SAT: spontaneous awakening trial; SBT: spontaneous breathing trial; IMS: ICU Mobility Scale; MRC-SS: Medical Research Council Sum-Score.

The primary objective of this work is to share essential evidence-based insights concerning the management and rehabilitation process of critically ill patients with obesity. Exploring a range of factors specific to this population, including general metabolic responses to critical illness, both non-invasive (NIV) and invasive mechanical (IMV) ventilatory support, the weaning process from IMV, and the assessment and functional rehabilitation processes performed by physiotherapists in the critical care setting, in collaboration with a multidisciplinary team. To summarize, key points related to these interventions are presented in Figure 1.

## Metabolic response to critical illness, the obesity paradox, and relevant clinical implications

During critical illness, the metabolic response to stress is viewed as an adaptive reaction to a life-threatening situation. This response is characterized by an alteration in the metabolic pathways that are most efficient in energy production and a shift in the utilization of energy substrates, which deviate from their normal self-regulation concerning their availability (8–10).

The impact and clinical consequences of this metabolic response to stress result in alterations in resting energy expenditure, hyperglycemia, loss of muscle mass, and issues related to mental health and cognitive functions. The loss of muscle proteins (muscle wasting) and their dysfunction (intensive care unit-acquired weakness [ICUAW]) are considered significant predictors of survival and comorbidities in the short and medium to long term for survivors of critical illness (10–15), respectively. Importantly, these effects are not exclusive to critically ill patients without obesity.

There are often uncertainties surrounding the optimal management of metabolic and organic support interventions for critically ill patients with obesity. It is estimated that between 28% and 36% of ICU admissions pertain to this population (16–20). Obesity itself is considered a clinical entity with a significant impact on various organs and systems, predisposing individuals to states of low-grade chronic inflammation, procoagulant tendencies, and insulin resistance. This strong correlation links obesity to the development of cardiometabolic diseases, a high incidence of morbidity and mortality, and a substantial reduction in the QoL (21).

However, in the context of acute illness (e.g., sepsis, moderate to severe acute respiratory distress syndrome, multi-organ failure.), it appears to have a “protective factor” against any of these situations, a phenomenon referred to as the Obesity Paradox (22–24). To date, there is a considerable heterogeneity in the available information regarding this concept. Systematic reviews and meta-analysis point toward a potential superiority in terms of protection and survival among critically ill patients with overweight and/or healthy metabolic obesity compared to those with sarcopenic obesity or a predominance of visceral adipose tissue accompanied by comorbidities, as well as compared to patients without obesity. The debate revolves around a significant difference in the number of patients with and without obesity admitted to ICUs, the variations in therapeutic approaches and clinical management for these patients compared to critically ill patients without obesity (e.g., a reduced administration of intravenous fluids), and the poor correlation that exists between BMI and the actual metabolic health of patients, taking into account the diverse phenotypes of muscle mass and adipose tissue distribution (22–25).

In general, it can be inferred that comprehensive trials are needed to investigate the energetic mechanisms at play during the different phases of critical illness, across various populations, both with and without obesity. This is crucial for obtaining reliable and context-specific information that can be reproducible in different settings. It appears that this protective state experienced by some individuals with obesity and healthy metabolic profiles is strongly associated with lower mortality, despite an increased prevalence of post-ICU comorbidities (19), and a higher utilization of resources and economic costs during the in-hospital stay (25).

Regarding the measurement of energy requirements, nutritional support, and metabolic care for muscle, international guidelines recommend the early initiation of enteral nutrition (within the first 24–48 h) over parenteral administration of micro and macronutrients. In critically ill patients, in the absence of indirect calorimetry, ASPEN 2016 recommends 11–14 kcal/kg of actual weight/day in patients with BMI of 30–50 and 22–25 kcal/kg of ideal weight/day in patients with BMI > 50. The recommended protein intake is 2.0 g/kg of ideal weight/day in patients with BMI of 30–40 and 2.5 g/kg of ideal weight in patients with a BMI > 40 (26–29).

It is essential to underscore the pivotal role that adequate nutritional support plays in promoting overall muscle health and influencing short and medium-term outcomes for critically ill patients. Nutrition stands as one of the tools available to healthcare professionals working in the ICU, contributing to enhancements QoL, reduced mechanical ventilation duration, shorter hospital stays, and decreased mortality rates (27, 30, 31). Furthermore, it is considered a fundamental prerequisite and a valuable adjunct for the effective implementation of timely mobilization programs within the ICU.

## Invasive ventilatory support in patients with obesity: structural and functional respiratory system alterations

Ventilatory support in patients with obesity can present challenges due to the structural and functional alterations in their respiratory system (32–34). These alterations may have a negative impact on the presence of critical illness. Some of these characteristics include reduced compliance of the respiratory system (Crs), expiratory reserve volume (ERV), functional residual capacity (FRC), and total lung capacity (TLC). It is estimated that there is a reduction in FRC of 5% to 15% for every 5 kg/m^2^ increase in BMI (35, 36). Furthermore, transthoracic and pleural pressures are increased, while transpulmonary pressure is reduced during spontaneous breathing. The adverse effects of these pulmonary changes include a tendency for atelectasis formation, cranial displacement of the diaphragm, airway collapse, air trapping, and alterations in the ventilation/perfusion (V/Q) relationship (35–38). These changes also manifest as hypoxemia and an increased respiratory workload (WOB) both at rest and during physical exercise.

Proper monitoring for the timely identification of air trapping and the presence of intrinsic positive end-expiratory pressure (PEEPi) during mechanical ventilation is crucial to avoid complications (32–34). This is achieved by observing and analyzing the flow/time curve on the mechanical ventilator’s graphical monitor, ensuring that expiratory flow reaches the baseline or zero line. When this does not occur, it is likely that the patient is experiencing air trapping, and a expiratory pause maneuver is necessary to measure PEEPi. Adjustments to the inspiratory-to-expiratory (I:E) ratio will facilitate an appropriate “emptying” of the tidal volume (TV) delivered by prolonging the expiratory time, which is highly beneficial in cases of obstructive airway problems (e.g., bronchospasm, dynamic hyperinflation, etc.).

All of these alterations, their assessment, and management should be considered by the critical care physiotherapist to ensure the optimal selection and safety of patients during functional interventions within an early mobilization program (EM) in the ICU.

## Ventilator-Induced Lung Injury, monitoring, and lung protection in patients with obesity

Ventilator-Induced Lung Injury (VILI) should be prevented in all patient populations. Nonetheless, critically ill obese patients demonstrate heightened elastance, particularly those with central fat distribution (android obesity), prompting a shift toward less rigorous lung protection objectives (32, 34).

Although acute respiratory distress syndrome (ARDS) is not the primary cause of acute respiratory failure requiring IMV in critically ill patients with obesity, it is still imperative to distinguish and make necessary adjustments in ventilatory settings and lung protection objectives (33). One of the most essential strategies involves accurately calculating the TV based on the patient’s ideal weight and gender. An initial TV ranging from 4 to 6 mL/kg of predicted body weight [PBW = 50 + 0.91 (cm of height – 152.4) in males, and PBW = 45 + 0.91 (cm of height−152.4) in females] is a suitable consideration for patients with ARDS, while patients without ARDS may benefit from a TV between 6 and 8 mL/kg of IBW [IBW for males = 23 × (height in meters)^2^ and IBW for females = 21.5 × (height in meters)^2^] (32). Notably, not all obese patients require a high positive end-expiratory pressure (PEEP). For those without ARDS, initial PEEP settings can vary between 5 and 10 cmH_2_O, based on BMI, as suggested by the LOV-ED study (39). However, patients with a BMI > 40 kg/m^2^ may require PEEP values exceeding 10 cm H_2_O. Some authors suggest the use of esophageal pressure balloon to guide PEEP titration, although it is not a standard practice. The adjustment of PEEP is not exclusively determined by pulmonary dynamics; instead, it is influenced by the interplay of other factors, including hemodynamic performance, enhancements in arterial gases, alveolar collapse, and overdistension (40). All of these factors should be taken into account during the evaluation and decision-making process.

Also, the relevance and clinical impact of other commonly used variables have been described and discussed. For example, driving pressure (dP) has not shown a significant impact on mortality in obese patients with ARDS (41). This, of course, does not mean that this population is exempt from developing VILI in any of its presentations. In general, a dP of < 17 cm H_2_O is recommended for patients with ARDS, and < 15 cm H_2_O for patients without ARDS (32, 34). Furthermore, adjusting plateau pressure relative to intraabdominal pressure (IAP) measured via a bladder catheter is suggested. Plateau pressure should be maintained below 27 cm H_2_O + (IAP – 13)/2 in patients with ARDS and 20 cm H_2_O + (IAP – 13/2) in patients without ARDS. It should be remembered that an increase in transpulmonary pressure is one of the main mechanisms of VILI, and in obese patients, elevated plateau pressure may be related to high transthoracic pressure rather than an increase in transpulmonary pressure with lung overdistension (34). Furthermore, some authors recommend maintaining a mechanical power of < 17–20 J/min, although its clinical utility is questionable (32).

Lastly, the prone position can be employed in critically ill obese patients receiving mechanical ventilation, provided that an experienced team is available (37). It is recommended to use a reverse Trendelenburg position to minimize the impact on the respiratory system, alleviate abdominal fat pressure, and prevent compression of thoracic organs (33, 34). Thus, to enhance diaphragm function and prevent cephalization, it is advisable for obese patients to assume sitting in bed, or recliner positions (semi-Fowler), as well as upright positions (42).

During early mobilization, adjustments in mechanical ventilation programming may be necessary to enhance exercise tolerance (43). For improved comfort and appropriate functional progress, conventional spontaneous ventilatory modes like pressure support (PSV) are recommended (44). Modifications in the fraction of inspired oxygen (FiO_2_) and/or pressure support (PS) may be implemented to reduce WOB, dyspnea, and perceived exertion during physical activity (43, 44). This is particularly noteworthy because of the heightened WOB, diminished exercise tolerance, and increased oxygen consumption (VO_2_) and serum carbon dioxide (CO_2_) levels commonly seen in this population as a result of obesity-related changes (45, 46).

A proper progression of mechanical ventilation will allow for early and timely weaning, thereby preventing pulmonary complications (33).

## Weaning process from invasive mechanical ventilation and non-invasive ventilatory support

The weaning process from IMV and NIV support can be a challenging task for clinical professionals when caring for critically ill obese patients, alongside the respiratory care provided during IMV (47, 48). There is a scarcity of evidence, established practices, and specific cutoff points for this weaning process. Nevertheless, some widely available and replicable guidelines within the ICU can serve as valuable references. These guidelines are outlined in Table 2 (47).

**TABLE 2:** Clinical guidelines and weaning predictors for discontinuing IMV before and during SBT.

Measurements/Predictors	Parameters before and during SBT
Consciousness state	GCS >12 points, awake and alert
Hemodynamic stability	Low doses of 1 or 2 vasoactive drugs, no myocardial ischemia, HR <130 bpm, no hypotension
Respiratory stability	Spontaneous breathing, FiO_2_ <60%; SpO_2_ >90% and no use of accessory muscles
Rapid Shallow Breathing Index (RSBI)	<60–105 breaths/min/L
Negative Inspiratory Force (NIF)	−20 to −30 cmH_2_O during SBT
Integrated Pulmonary Index (IPI)	>8 points
Effective cough	Peak Expiratory Flow (PFE) >−60 L/min
Type of Spontaneous Breathing Trial (SBT)	Avoid T-piece and PEEP/PS 0,0 cmH_2_O
PEEP	>5 cmH_2_O, preferably 8 cmH_2_O during SBT
Pressure Support (PS)	<8 cmH_2_O during SBT
Diaphragmatic excursion (diEx)	>1.8 cm during SBT
Diaphragmatic Thickening Fraction (diThF%)	30–36% during SBT Note: Difficulty in obtaining measurements may be encountered in this population

SBT, Spontaneous breathing trail; GCS, Glasgow Coma Scale; HR, heart rate; FiO2, fraction of inspired oxygen; RSBI, Rapid Shallow Breathing Index; NIF, Negative Inspiratory Force; diEx, diaphragmatic excursion; diThF, diaphragmatic thickening fraction.

Initially, the implementation of appropriate sedation and analgesia protocols allows for timely suspension, enabling the patient to transition to spontaneous breathing. Prolonged periods of deep sedation and diaphragmatic inactivity have a strong correlation with increased duration of invasive mechanical ventilation, extended hospital stays, and higher mortality rates. It is recommended to avoid using benzodiazepines due to the risk of their accumulation in adipose tissue, resulting in prolonged release and excretion (49). Moreover, their use is associated with an increased risk of developing delirium, which directly impacts significant clinical outcomes, including mortality.

Regarding the level of respiratory support during the spontaneous breathing trial (SBT), there is a degree of controversy. It is recommended to conduct the SBT with a minimum of 5 cmH_2_O of PEEP and PS. Using a T-piece or setting PEEP and PS to 0.0 cm H_2_O, especially in patients with a BMI greater than 35 kg/m^2^, should be avoided. This is because it can potentially result in a significant increase in the WOB, which may lead to extubation failure (46, 50, 51). Mahul et al. demonstrated that these two SBT methods can predict post-extubation respiratory effort (51). Furthermore, the development of atelectasis often plays a role in extubation failure, leading to higher rates of reintubation and prolonged IMV (34, 42, 45).

Post-extubation care includes: placing the patient in an upright seated position, using NIV and high-flow oxygen therapy (HFNC) to prevent reintubation in high-risk individuals. Post-extubation NIV in obese patients has demonstrated benefits, such as improved oxygenation, homogenous pulmonary ventilation distribution, and a reduction in the pendelluft effect. Moreover, it has been associated with a lower risk of reintubation and decreased mortality (34, 45, 52).

## Non-invasive ventilatory support

The use of non-invasive devices for ventilatory support in obese patients admitted to the ICU is highly beneficial for the management and prevention of respiratory complications. The choice of the device will depend on each patient’s clinical presentation and diagnosis (33, 34, 52). Within the literature, options range from conventional oxygen therapy to HFNC and NIV. In addition, and as mentioned previously, the use of HFNC and NIV can help prevent intubation and reduce complications following extubation (33, 45, 52).

Compared to conventional oxygen therapy devices, HFNC appears to offer greater benefits for alveolar ventilation. It reduces dead space, improves oxygenation, and lowers the WOB when properly used. High-flow therapy provides continuous airway pressure, which can lead to slight lung recruitment, although further evidence is required on this matter (53).

On the other hand, NIV provides benefits for obese patients by enhancing ventilatory mechanics and FRC (50, 54). This is attributed to the delivery of continuous positive airway pressure and pressure support. It also improves lung aeration, thus enhancing patient oxygenation, while reducing the risk of developing atelectasis and lowering the WOB (45). In patients experiencing acute pulmonary edema due to positive pressure withdrawal (WiPO), the use of NIV is strongly recommended (48, 52).

At present, it remains uncertain whether HFNC or NIV holds a superiority in the management of obese patients across different clinical scenarios. Systematic reviews and meta-analyses suggest similar outcomes in critically ill post-extubation patients (55). Hence, their utilization should be based on the resource availability at each hospital facility.

The recommended parameters for NIV include a PS to achieve a TV between 6 and 8 mL/kg of ideal weight, a respiratory rate (RR) under 30 breaths per minute, PEEP between 5 and 10 cmH_2_O and oxygen saturation (SpO_2_) above 90% (33, 54). Close monitoring is imperative for the prediction of extubation failure or the need of intubation. Hence, clinical monitoring to estimate WOB include increased RR and the use of accessory muscles. Additionally, the use of scales such as iROX and HACOR score should not be overlooked (56, 57).

Given the multitude of potential complications and unfavorable outcomes associated with intubating or reintubating critically ill patients with obesity, preventing intubation or reintubation in these patients is of paramount importance. Nevertheless, early recognition of non-invasive device failure is crucial to prevent delayed intubation and reduce patient mortality (33, 34).

## Early (Timely) Mobilization in critically ill patients with obesity

EM in critically ill obese patients is a commonly used intervention to prevent functional impairments resulting from their critical condition and ICU stay. It involves introducing physical exercise within the first 2 to 5 days after admission to the ICU. However, recently, the concept of “timely mobilization” has gained prominence (58–60).

**FIGURE 2:**
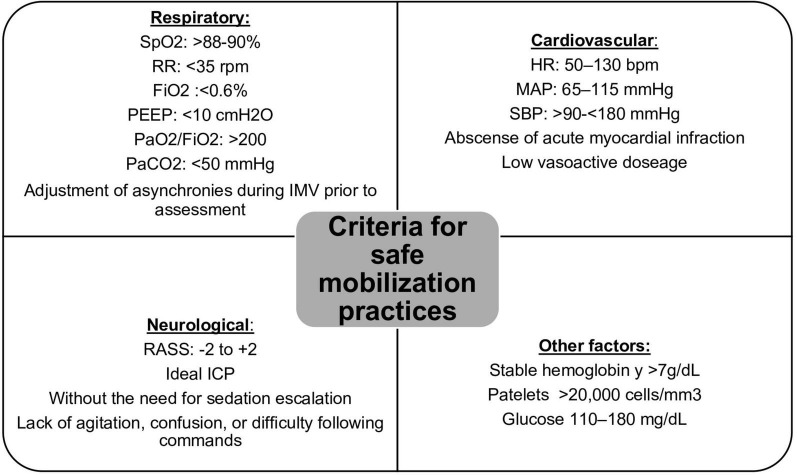
Safety criteria for EM. SpO2, partial oxygen saturation; RR, respiratory rate; FiO2, fraction of inspired oxygen; PEEP, positive end-expiratory pressure; PaO2, arterial oxygen pressure; PaCO2, arterial carbon dioxide pressure; HR, heart rate; MAP, mean arterial pressure; SBP, systolic blood pressure; RASS, Richmond Agitation-Sedation Scale; ICP, intracranial pressure.

One of the most crucial factors for ensuring the positive impact of EM is conducting it within the first 7 days of ICU admission (61). To achieve this, patients must undergo continuous and systematic assessments to identify the optimal moment for effective and timely physical exercise. Patient safety is always a top priority. It’s worth noting that criteria for clinical stability, such as hemodynamic, respiratory, neurological, and metabolic factors, can often be met through the use of extracorporeal support devices like IMV, extracorporeal membrane oxygenation (ECMO), or medications with vasopressor and inotropic properties (62, 63). These factors do not necessarily exclude the possibility of implementing an EM program. This collaborative approach, combined with ongoing training, clear communication channels, and teamwork, will aid in achieving the intended goals. Figure 2 outlines the general criteria for safe mobilization practices (64, 65).

Obesity is not a contraindication for EM, but it can pose a challenge for the involved healthcare personnel and the execution of the practice. The multidisciplinary team should seek strategies to minimize immobility in these patients (63, 66).

Currently, the prevailing approach to EM involves functional physical exercise. Existing evidence emphasizes the importance of focusing on patients’ mobility and functional abilities over other forms of physical progression. Recent research suggests that shorter intervention periods are strongly linked to reduced time on IMV and shorter stays in the ICU (67).

Careful planning to determine the level of functionality to which this patient group will be progressed is crucial for both patient and staff safety. Therefore, the use of scales designed to assess patients’ mobility and functionality within the ICU is essential. These scales not only assist in predicting hospital discharge but also exhibit a moderate correlation between the ICU Mobility Scale (IMS) and the Medical Research Council Sum-Score (MRC-SS), making the IMS a potential surrogate for muscle strength (68, 69).

Prescribing physical exercise should be done judiciously, based on the functional challenges determined for each patient, taking into account their pre-ICU and current level of mobility (68). These challenges can be graded by complexity and referred to as functional milestones, which may include activities like rolling in bed, sitting on the edge of the bed, standing, static marching, assisted walking, and independent walking (67). Proper patient positioning plays a crucial role in preventing respiratory complications, such as atelectasis and increased air trapping, and can positively impact ventilatory function in obese patients by improving thoracoabdominal movement and chest wall diameter changes. Also, the utilization of assistive devices for sitting, standing and walking should be taken into account in this population. Prior to their use, the physiotherapist must verify the weight limits of each of these additions. As mentioned previously, the use of NIV support devices, such as HFNC and/or NIV, in spontaneously breathing obese patients, can be considered as a safe alternative to improve exercise tolerance and physical endurance (43, 44, 46). This approach may contribute to the patient’s functional progress toward higher levels of mobility.

We strongly recommend conducting a pre-EM assessment that includes identifying barriers to mobilization, determining the required staff, assessing available accessories, ensuring the presence of the necessary equipment and instruments for airway management in case of emergencies, and always having a contingency plan in place (70). It’s essential to be aware that many obese patients may have a difficult airway. Therefore, having access to a videolaryngoscope during functional progression becomes imperative to enhance safety (34). In patients with a BMI > 40 kg/m^2^, precautions should be heightened, and a cost-benefit analysis of the intervention should be conducted to identify the ideal time to progress patient’s functionality.

EM sessions with very high levels of mobility or intensity and longer duration can lead to an increased occurrence of adverse events (71). The application of these same principles appears to be prudent in obese patients. The guidelines for critical care physiotherapists attending to this population involve aiming to get the patient out of bed as soon as possible, promoting verticalization, and engaging in functional physical exercise (59, 72). However, underestimating the patient’s physical capacity can lead to an inadequate exercise dosage that may result in poor functional outcomes.

It is worth noting that having knowledge of the patient’s pre-ICU functional status is highly valuable. This allows us to set more realistic goals during interventions within the ICU, tailored to the patient’s needs and enabling them to achieve their maximum level of functionality. Cultural shifts are required to enhance the care of these patients. Interventions in this population are becoming increasingly common and it is a necessity for us to be prepared (65).

## Psychological and emotional state

The lack of infrastructure within healthcare facilities and the increased workload in serving this patient population can generate a certain degree of discomfort (73). It is the responsibility of healthcare professionals to promote greater education and preparedness for addressing these patients. Additionally, specific initiatives should be developed to raise awareness and overcome the social discomfort in the care of patients with obesity (74). Also, motivation will play an important role that can help with adherence to EM programs and post-ICU rehabilitation. Finally, it is essential to have and manage appropriate infrastructure and materials for critically ill obese patients, as they are and will continue to be a significant part of our intensive care practices.

## Conclusion

Obesity, as a clinical condition, is a health issue affecting approximately one-fifth of the population in the ICU. The predisposition to numerous complications related to hospitalization and their impact on multiple organs and systems seems to be mitigated in individuals with metabolically healthy obesity phenotypes. However, disability rates and resource utilization in this population remain higher compared to individuals without obesity. Rehabilitating critically ill obese patients presents logistical challenges for critical care physiotherapists and the rest of the team involved in early mobilization programs. Understanding anticipated standards, continuous training, and effective lines of thought and communication will equip clinicians with the necessary tools to improve clinical outcomes such as quality of life, functionality, return to economic activities, and mortality in this population.

## Author contributions

MM-C: Conceptualization, Visualization, Writing−review and editing. RJ-B: Writing−original draft, Writing−review and editing. AG-G: Project administration, Writing−review and editing. DM-H: Writing−review and editing. DL-G: Conceptualization, Writing−review and editing. AM-V: Conceptualization, Writing−review and editing. CN-R: Writing−review and editing. JD-C: Conceptualization, Writing−review and editing.
